# CDK12 and CDK13 in oncology: from RNA regulation to therapeutic targeting

**DOI:** 10.1007/s13402-025-01131-z

**Published:** 2026-01-05

**Authors:** Julia Dudkiewicz-Garbicz, Paweł K. Włodarski

**Affiliations:** 1 Department of Histology and Embryology, Medical University of Warsaw, Warsaw, Poland; 2 Harvard Medical School, Boston, MA USA; 3 Department of Pediatric Oncology, Dana-Farber Cancer Institute, 450 Brookline Avenue, Boston, MA 02215 USA

**Keywords:** CDK12, CDK13, RNA polymerase II, DNA damage response, Kinase inhibitors, Targeted cancer therapy

## Abstract

This review aims to synthesize current knowledge on Cyclin-dependent kinase 12 (CDK12) and Cyclin-dependent kinase 13 (CDK13), two transcriptional kinases with distinct and overlapping roles in gene regulation, genome stability, and cancer biology. These kinases control transcription elongation and co-transcriptional processing, including splicing and polyadenylation, thereby linking RNA polymerase II activity with DNA repair and replication stress response. CDK12 and CDK13 play context-specific roles across malignancies, where amplification, truncating mutations, fusions, and deletions variably alter their transcriptional output and downstream DNA repair programs. Their dysregulation contributes to oncogenesis, metastasis, and resistance to conventional therapies. There is growing therapeutic interest in CDK12/13 inhibition, especially as selective inhibitors of single DNA repair nodes have shown limited success. Selective CDK12/13-targeting agents have shown efficacy in preclinical models by inducing synthetic lethality, restoring sensitivity to PARP inhibitors, and are being explored to potentiate immune checkpoint blockade. This review highlights the biological rationale for targeting CDK12 and CDK13 in cancer, summarizes emerging therapeutic strategies, and identifies key opportunities for integrating these approaches into precision oncology.

**Graphical Abstract:**

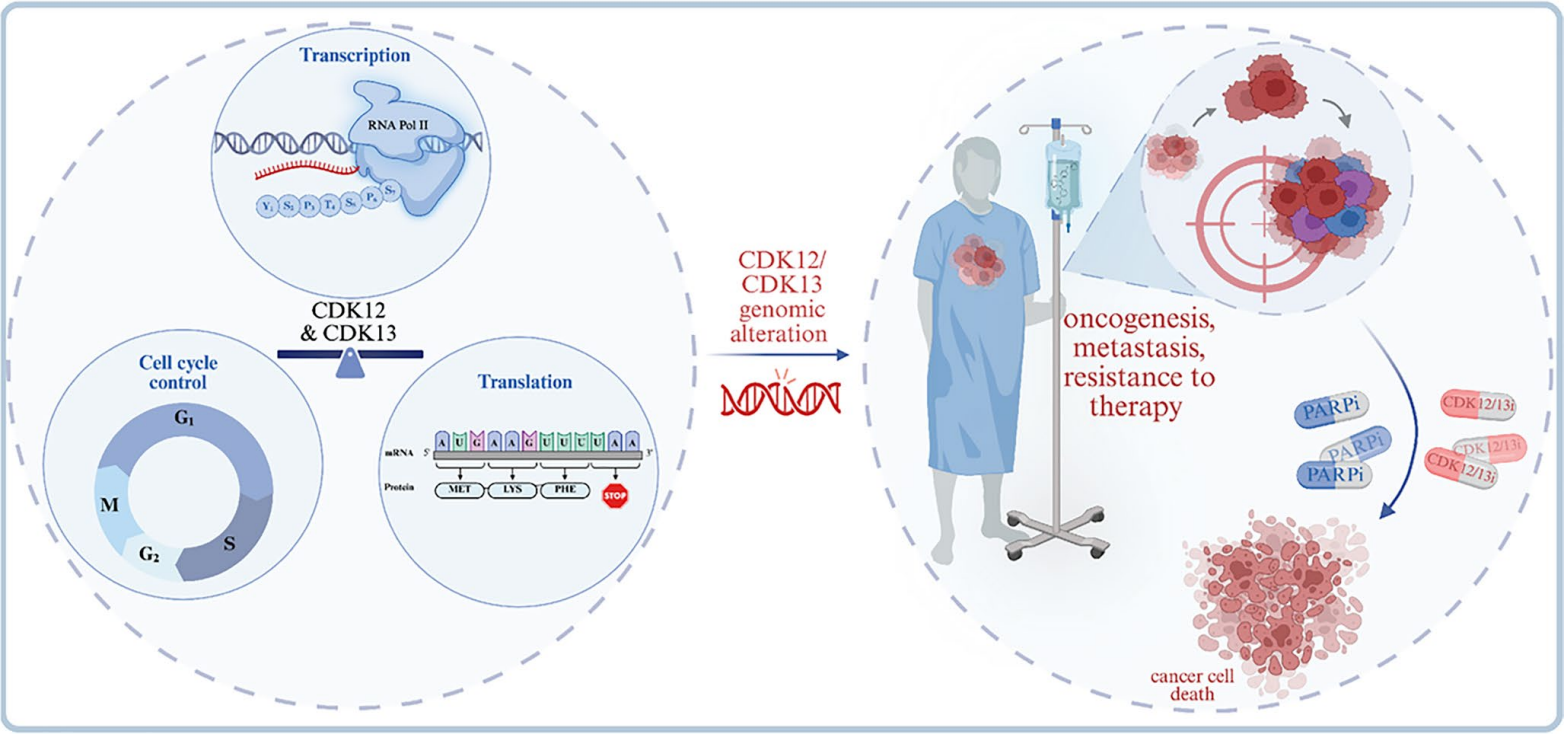

**Supplementary information:**

The online version contains supplementary material available at 10.1007/s13402-025-01131-z.

## Introduction

Dysregulated transcription is a hallmark of cancer, driving aberrant gene expression programs that sustain proliferation, genomic instability, and therapy resistance [1]. Among the transcriptional regulators that coordinate these oncogenic networks are cyclin-dependent kinases 12 and 13 (CDK12 and CDK13), which, in complex with cyclin K, phosphorylate the C-terminal domain (CTD) of RNA polymerase II (Pol II), coupling transcription elongation with RNA processing, DNA repair, and replication stress responses [2–6].

Genomic alterations of CDK12 and CDK13, including amplifications, truncating mutations, fusions, and deletions, occur across malignancies and can yield opposite phenotypes depending on context: amplification or overexpression enhances oncogenic transcriptional programs, whereas loss-of-function disrupts DNA damage response (DDR) gene expression and promotes genomic instability [7–10]. CDK12 amplification frequently occurs in HER2-positive breast and gastric cancers, where it enhances PI3K-AKT and WNT signaling [11–14]. In contrast, truncating mutations in ovarian and prostate cancers produce tandem-duplication-rich genomes that define an immunogenic subset of advanced prostate cancer [9, 10].

Mechanistically, CDK12/13 selectively sustain processive transcription of long, multi-exonic DDR and cell-cycle genes (e.g., *BRCA1*, *ATR*, *FANCD2*, *RAD51*). Their inhibition or loss diminishes DDR protein abundance, increases replication stress, and triggers double-strand breaks, functionally mimicking BRCA1/2 loss [2, 5, 15, 16]. However, emerging evidence indicates that the cellular consequences of CDK12 dysfunction depend on temporal context: while acute inhibition leads to abrupt downregulation of homologous recombination genes and DNA damage accumulation, chronic genetic loss can trigger adaptive transcriptional rewiring that partially restores DNA repair capacity. In tumors chronically adapted to CDK12 deficiency, most homologous recombination genes regain expression through compensatory mechanisms, including partial functional compensation by CDK13, while specific loci such as *ATM* remain persistently downregulated. This adaptive equilibrium may explain the limited clinical efficacy of PARP inhibitor monotherapy in some CDK12-mutant cancers and underscores the need for combinatorial or dual CDK12/13-targeted approaches [17].

CDK12 loss also defines immunogenic tumor subsets characterized by tandem duplications, gene fusions, and increased neoantigen production, predicting responsiveness to immune checkpoint blockade [10, 18]. Inhibitors and degraders targeting CDK12/13 suppress expression of DDR genes, sensitize tumors to PARP inhibitors, and enhance sensitivity to DNA-damaging agents [19–21].

This review synthesizes current knowledge on CDK12 and CDK13 as transcriptional kinases at the intersection of RNA metabolism, genome maintenance, and oncogenic signaling. We focus on their dual roles as oncogenes and tumor suppressors, summarize the molecular consequences of their dysregulation across cancer types, and highlight recent advances in the development of selective inhibitors and targeted degraders with translational potential in precision oncology.

## Physiological roles of CDK12 and CDK13

**Fig. 1:**
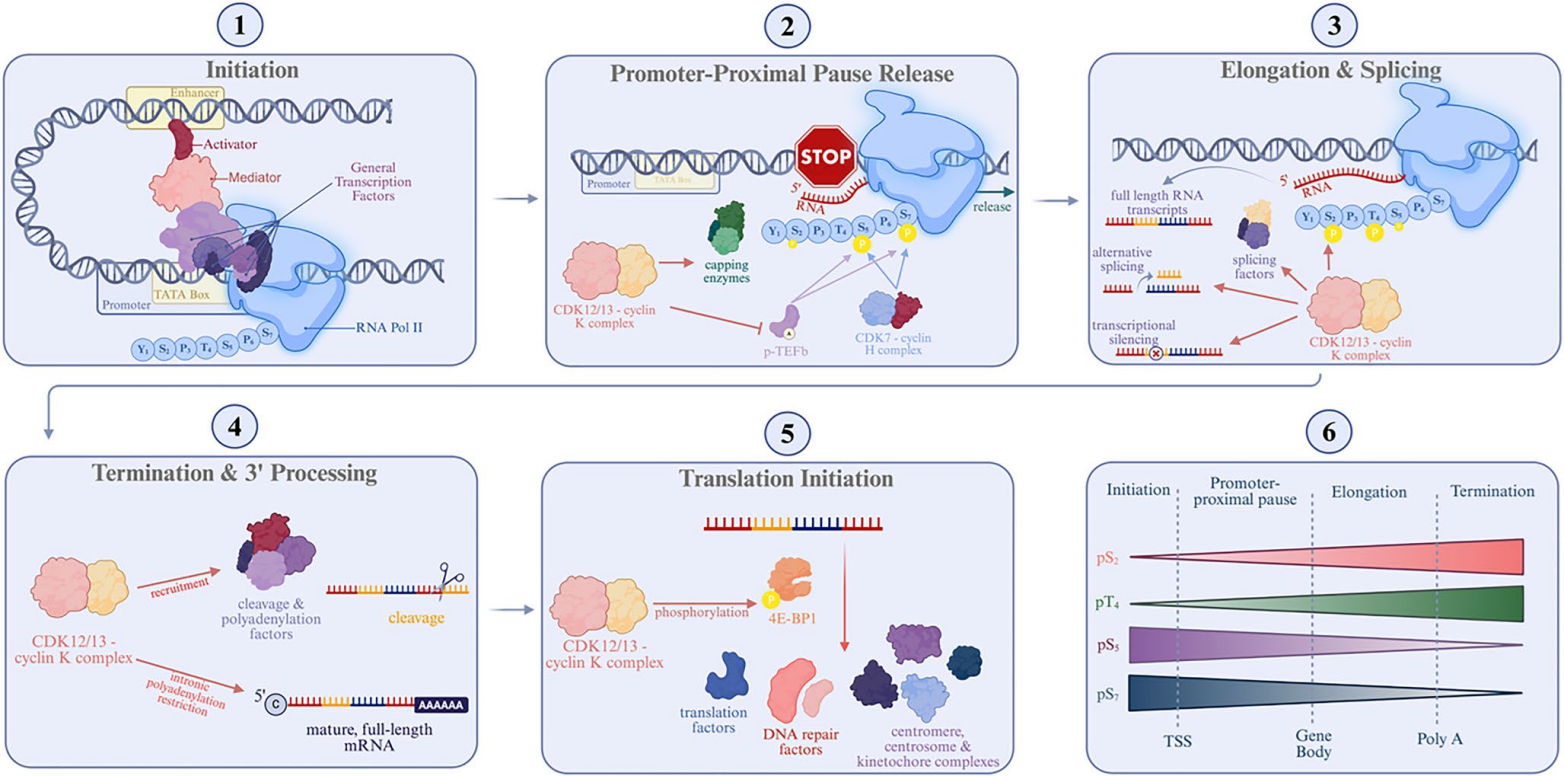
The CDK12/13 complexes regulate multiple stages of transcription and translation. 1. Initiation: RNA Polymerase II, together with general transcription factors, activators, and the mediator complex, assembles into the pre-initiation complex (PIC). Following PIC formation, Pol II transcribes a short RNA before pausing at the promoter-proximal pause site, allowing for quality control prior to elongation. 2. Promoter-Proximal Pause Release: The CDK7-cyclin H complex and p-TEFb (CDK9-cyclin T complex) promote pause release and productive elongation. CDK12/13-cyclin K complex represses p-TEFb. 3. Elongation and Splicing: During productive elongation, CDK12/13 phosphorylate Ser2 of Pol II CTD, enhancing elongation rate and processivity to ensure efficient synthesis of full-length transcripts. CDK12/13 also regulate the activity and expression of splicing factors. Modulation of Pol II elongation rate influences alternative splicing outcomes. 4. Termination and 3’ Processing: CDK12 promotes recruitment of cleavage and polyadenylation factors, ensuring proper 3’ mRNA processing. CDK12/13 prevent premature transcription termination, maintaining transcriptional fidelity. 5. Regulation of translation initiation: CDK12/13 promote translation by phosphorylating and displacing 4E-BP1 from mRNA targets, which facilitates recruitment of translation factors to the 5′ cap. 6. Pol II CTD phosphorylation dynamics: As transcription progresses, phosphorylation of Ser5 and Ser7 decreases, whereas phosphorylation on Ser2 and Thr4 increases. Adapted from “Eukaryotic Gene Regulation - Transcriptional Initiation”, by BioRender.com (2025)

CDK12 and CDK13 are transcriptional kinases that, together with cyclin K, coordinate multiple stages of gene expression, thereby maintaining genome stability and cell-cycle control [5, 22]. While sharing overlapping roles in transcriptional regulation, CDK12 preferentially sustains DNA damage response (DDR) gene expression, whereas CDK13 more strongly influences splicing and RNA processing networks [23–25]. In cancer, disruption of these activities contributes to transcriptional imbalance, replication stress, and therapeutic vulnerabilities. The principal molecular functions of these kinases are summarized in Fig. 1.

### Transcription

#### C-Terminal domain of RNA polymerase II: a regulatory hub

Transcription requires precise cooperation among Pol II, transcription factors, cofactors, chromatin remodelers, and CTD-modifying enzymes [26, 27]. The heptapeptide repeats of the Pol II CTD undergo dynamic post-translational modifications, mainly phosphorylation, creating a platform that coordinates transcription with co-transcriptional RNA processing [28, 29]. Both CDK12 and CDK13 phosphorylate CTD Ser2 and Thr4 to promote elongation, processivity, and alternative polyadenylation control in a gene-specific manner [3, 24]. Under cancer-associated stress conditions, increased CTD phosphorylation by CDK12/13 sustains oncogenic transcriptional programs and maintains survival under replication stress [14, 30].

#### Transcription initiation & promoter-proximal pausing

Pol II recruitment to promoters initiates transcription (Fig. 1). The unphosphorylated CTD interacts with initiation factors and mediator, forming the pre-initiation complex [24]. After promoter clearance, Pol II synthesizes a short RNA (≈25–50 nt) before pausing at the promoter-proximal site. This checkpoint ensures proper capping and regulatory control [23]. This mechanism is particularly relevant in cancer, where altered pausing dynamics driven by hyperactivated transcriptional kinases contribute to uncontrolled gene expression and stress-response activation.

#### Promoter-proximal pause release and elongation

Pause release depends on phosphorylation of CTD Ser5/Ser7 by CDK7-cyclin H and CDK9-cyclin T (p-TEFb) [24]. CDK12/13 functionally represses p-TEFb [18]. Once Pol II is released and elongation begins, CDK12/13 phosphorylate CTD Ser2, increasing Pol II processivity and ensuring full-length mRNA synthesis [5, 30]. Under oncogenic signaling, this process enables rapid expression of stress-response and DDR genes, sustaining proliferation and therapy resistance [2, 16].

#### Splicing regulation and transcriptional coupling

CDK12/13 couple Pol II elongation with splicing by phosphorylating CTD Ser2 and recruiting splicing factors (Fig. 1) [31, 32]. Their RS-rich domains serve as docking sites for splicing regulators [33, 34]. Depletion of either kinase disrupts RNA processing, emphasizing their role in coordinating elongation with spliceosome activity [4, 5]. In tumor cells, aberrant CDK12/13 activity contributes to induction of alternative splicing that supports oncogenic signaling and therapy resistance [35].

#### 3’ end processing - polyadenylation and cleavage

CDK12-dependent CTD Ser2 phosphorylation recruits cleavage and polyadenylation factors to nascent transcripts, which are normally involved in transcript termination at the 3’ end (Fig. 1) [16, 36, 37]. Loss of CDK12 disturbs elongation and Pol II processivity, which triggers premature intronic polyadenylation (IPA), generating truncated transcripts [9, 10]. CDK13 similarly suppresses premature termination by limiting IPA usage [5, 38]. In cancer, many key DDR genes (e.g., *BRCA1*, *ATR*) are unusually long and contain numerous cryptic polyadenylation sites, so loss of transcript integrity in these genes leads to homologous recombination deficiency and renders tumors more sensitive to PARP inhibitors and platinum-based therapy [21, 39, 40].

### Translation and cell cycle

CDK12 also regulates translation of genes encoding DDR and mitotic proteins [41]. It phosphorylates 4E-BP1 at Ser65/Thr70, promoting translation factor recruitment and synthesis of proteins required for chromosome segregation and genome maintenance [14, 42]. CCNK also regulates phosphorylation of cyclin E1 on Ser366, preventing CDK2 activation and controlling G1/S transition [43]. Aberrant CDK12/13 signaling enhances translation of oncogenic transcripts, linking transcriptional control to protein synthesis reprogramming in cancer [44]. Loss of CDK12/13 or cyclin K disrupts pre-replicative complex formation, causing G1 arrest and DNA-damage accumulation [4, 5]. Persistent replication stress in CDK12-deficient cells contributes to chromosomal instability, a recognized driver of tumor evolution and chemoresistance [45].

### CDK12/13 in embryonic cells and human development

CDK12 and CDK13 are indispensable for embryogenesis. *Cdk12* ^-/-^ mice exhibit lethal genomic instability due to impaired DDR-gene expression [37]. Both kinases maintain pluripotency in embryonic stem cells by preserving transcriptional fidelity [46]. CDK12 and CDK13 also regulate neurogenesis and axonal extension; their loss induces microcephaly and neurodegeneration in animal models [47, 48].

In humans, heterozygous CDK13 mutations in the kinase domain cause developmental delay, structural cardiac anomalies, seizures, corpus callosum abnormalities, and craniofacial dysmorphism [49]. A recent clinical case report suggests a potential for increased cancer predisposition in individuals with germline CDK13 alterations. Documented cases include pediatric malignancies such as Acute Lymphoblastic Leukemia in a 9-year-old boy, and the presence of dysplastic nevus in an adolescent [50, 51].

## The dual role of CDK12 and CDK13 in cancer

**Fig. 2:**
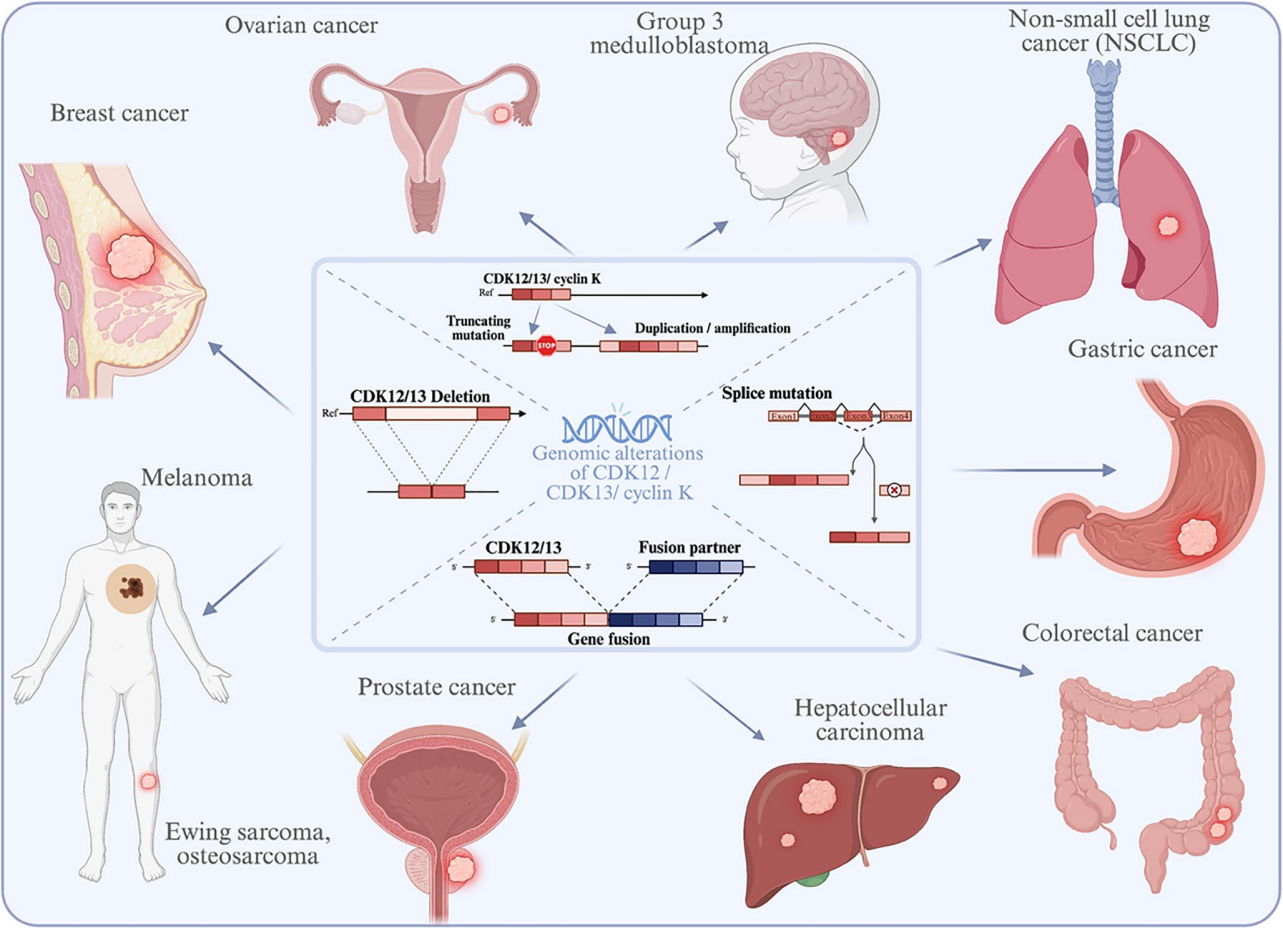
Diverse genomic alterations of *CDK12/13/CCNK* in cancer. These alterations can either drive oncogenic activity (e.g., amplifications) or lead to a tumor suppressor phenotype (e.g., deletions and truncating mutations). Representative alteration frequencies observed in key malignancies include: CDK12 truncation/loss-of-function in approximately 6% of metastatic prostate cancers, 2–3% of high-grade serous ovarian cancers [21], and CDK12 recurrent somatic alterations (bi-allelic deletions, genomic amplifications and mutations) in 13% of breast cancers [52]. Created with BioRender.com

Gene expression dysregulation is a defining hallmark of tumorigenesis [1]. The transcriptional kinases CDK12 and CDK13 are central players in this process, but their influence is highly context-dependent, acting as oncogenic drivers in some tumors and tumor suppressors in others. Fig. 2 summarizes the principal types of genomic alterations affecting the CDK12/13-cyclin K complex and the tumor contexts in which they are most frequently observed. These include gene amplifications, deletions, truncating mutations, and fusion events that can either enhance oncogenic transcriptional output or compromise DDR fidelity. Such diversity in alteration type underlies the dual functional spectrum of CDK12/13 across human cancers, discussed in the following sections.

**Fig. 3:**
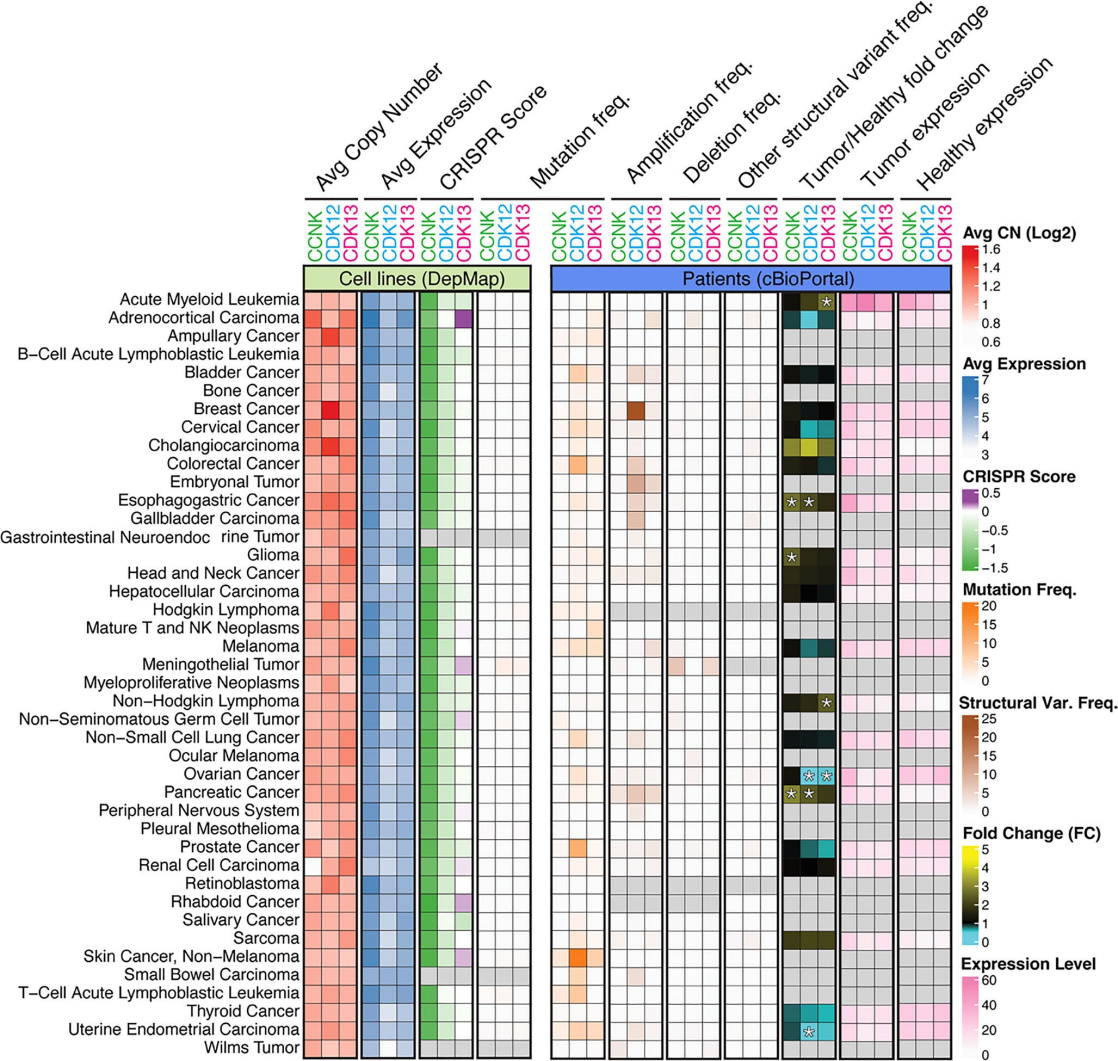
Genomic and transcriptomic features of *CCNK*, *CDK12*, *CDK13* across human cancers. This figure integrates multi-omic data from cell line models (left panel) and patients (right panel) to provide a comprehensive pan-cancer landscape. Data was integrated from publicly available datasets (DepMap 24Q4 and cBioPortal, 231 studies, Supplementary Table 1) and harmonized using a standardized ontology to map disparate histological subtypes to unified cancer lineages (rows). We aggregated lineage-specific means for WGS copy number, gene expression, and CRISPR Chronos scores, alongside mutation frequencies (mutated/total cell lines). Additionally, tumor versus normal expression comparisons were integrated from GEPIA, utilizing its standard one-way ANOVA on log₂(TPM + 1) values, where TMP is Transcripts Per Million. Asterisks (*) denote statistically significant tumor-to-normal expression differences (*p* < 0.05), and grey squares indicate missing data values. These multi-omics metrics for *CCNK, CDK12*, and *CDK13* were merged and visualized using the ComplexHeatmap R package

To fully appreciate the diverse roles of CDK12 and CDK13 in oncology, it is essential to contextualize their alterations within the broader pan-cancer landscape. The selective pressure for tumors to co-opt these kinases is not uniform; rather, it manifests through distinct genomic, transcriptomic, and dependency patterns that vary by malignancy. Integration of large-scale, multi-omic datasets from the DepMap project and cBioPortal reveals a comprehensive overview of this landscape (Fig. 3) [53–57].

This analysis reveals several key trends. First, a striking functional dependency on both CCNK and CDK12 is evident across most cancer cell lines, as demonstrated by pan-cancer negative CRISPR scores. This dependency co-exists with widespread transcriptional upregulation. While this overexpression is a general feature, specific genomic events are concentrated in particular tumor types. For instance, CDK12 alterations are notably frequent in breast, colorectal, pancreatic and prostate carcinomas. Interestingly, very few cancer cell lines harbor CDK12/13 mutations or deletions, which points to a possible reduced fitness of such clones when cultured in vitro. This is consistent with the idea that complete loss of CDK12/13 function is poorly tolerated outside specific genetic backgrounds. While comprehensive functional dependency and expression data are widely available for cell lines (DepMap), the mutation burden from patient cohorts (cBioPortal, Supplementary Table 1) is missing for some of the malignancies (represented by the gray squares in Fig. 3), reflecting current gaps in systematic genomic characterization for specific cancer types.

### The oncogenic roles of CDK12/13

In many cancers, elevated CDK12/13 activity is a key driver of tumor progression. This is frequently achieved through gene amplification or transcriptional upregulation (Fig. 3) [8]. Analysis of tumor versus normal tissue shows that *CDK12* and *CDK13* are frequently upregulated across multiple cancer types (Fig. 3) [7]. Enhanced CDK12/13 activity drives oncogenic transcriptional programs, sustaining higher expression levels of replication and stress-response genes and supporting tolerance to replication stress [25, 41]. In breast cancer, overexpression of CDK12 activates the WNT/β-catenin and PI3K–AKT pathways [41], while in gastric cancer CDK12 cooperates with PAK2 to sustain MAPK signaling [25].

Large-scale CRISPR dependency screens confirm that numerous tumor cell lines display strong dependence on CDK12/13 [20] (Fig. 3). These data position CDK12/13 among key transcriptional kinases that maintain oncogenic gene expression networks and indicate that their inhibition may disrupt the transcriptional addiction characteristic of numerous cancers [1].

### Tumor suppressor functions, genomic instability, and adaptive responses to CDK12/13 loss

In contrast to their oncogenic roles, CDK12 and CDK13 can also function as tumor suppressors in specific contexts, particularly in ovarian and metastatic castration-resistant prostate cancers [9, 10]. In these tumors, *CDK12* is frequently inactivated by truncating mutations, deletions, or gene fusions (Fig. 3). Loss of *CDK12* disrupts transcription of key DDR genes, such as *BRCA1*, *ATR*, and *FANCI*, and leads to defective HR. The resulting “BRCAness” phenotype manifests as tandem duplications and widespread genomic instability [2, 16]. Mechanistically, *CDK12* loss promotes transcription-replication conflicts, causing polymerase collisions and DNA double-strand breaks [6]. This triggers a dependency on compensatory repair pathways and sensitizes cells to PARP inhibitors, representing a prototypical synthetic-lethal interaction [19, 39].

However, the biological consequences of CDK12 inactivation differ between acute pharmacologic inhibition and chronic genomic loss. In tumors chronically adapted to CDK12 deficiency, HR genes can become transcriptionally restored through compensatory mechanisms, including partial redundancy with CDK13, while specific loci such as *ATM* remain truncated and downregulated. As a result, these tumors often retain functional HR capacity and lack the genomic scars typical of HR-deficient cancers [17]. This adaptive equilibrium may explain the limited clinical efficacy of PARP inhibitor monotherapy in certain CDK12-mutant malignancies and underscores the importance of co-targeting CDK13 or other transcriptional dependencies to overcome resistance.

### CDK12 and tumor immunogenicity

The loss of CDK12 function profoundly impacts tumor immunogenicity through multiple, interconnected mechanisms. Primarily, the resulting genomic instability leads to the accumulation of tandem duplications, which generate a high burden of fusion-derived neoantigens [9, 10]. This enhanced neoantigen load increases the tumor’s visibility to the immune system, often fostering a T-cell-inflamed microenvironment characterized by elevated expression of interferon-stimulated genes and pro-inflammatory cytokines [58].

Beyond increasing antigen presentation, CDK12 directly shapes immune evasion pathways. It has been shown to regulate the alternative splicing of CD274 (encoding PD-L1), thereby controlling the abundance of specific immunomodulatory isoforms at the cell surface [59].

Furthermore, the impaired DDR inherent to *CDK12* loss can lead to the accumulation of DNA breaks. This damage can trigger the release of cytoplasmic DNA, activating the cGAS/STING innate immunity pathway and further influencing the tumor-immune landscape [60]. Bao et al. recently reported that CDK12/13 inactivation causes transcription-replication conflicts, cytosolic DNA release, and activation of STING and TBK1, which promoted CD8^+^ T cell anti-tumor response [61].

These findings establish CDK12/13 as regulators of tumor-immune interactions, whose genetic or functional status may serve as a predictive biomarker for response to immune checkpoint blockade [58].

## The role of CDK12/13 across cancer types

The genomic and functional alterations of CDK12 and CDK13 exhibit remarkable context specificity across malignancies (Figs. 2–3). Their dysregulation can enhance transcriptional output of oncogenic programs or, conversely, disable DNA-repair transcription, producing distinctive vulnerabilities that can be therapeutically exploited. The following subsections outline cancer-type-specific patterns of CDK12/13 alteration, functional consequences, and pharmacologic implications.

### Metastatic castration-resistant prostate cancer

*CDK12* alterations occur in roughly 7% of metastatic castration-resistant prostate cancers (mCRPC) and are predominantly biallelic truncating mutations that disrupt the kinase domain [10]. These lesions define a transcriptionally distinct subtype characterized by tandem duplications, genomic instability, and increased neoantigen burden, resulting in a partially inflamed tumor microenvironment [10, 58]. While CDK12 typically functions as a tumor suppressor, its loss drives context-dependent outcomes. In prostate cancer organoids, CDK12 inactivation enhances androgen-receptor (AR) signaling and induces enzalutamide resistance, whereas combined inhibition of CDK12/13 (THZ-531) and AR blockade (bicalutamide, apalutamide, or enzalutamide) exerts synergistic cytotoxicity [18].

*CDK12* loss promotes DNA-damage accumulation and p53 activation, which constrains tumor growth unless TP53 is co-inactivated, which is a frequent event that bypasses this checkpoint and accelerates progression [10, 18]. *CDK12*-deficient tumors exhibit strong cytokine induction and T-cell infiltration, consistent with the immunogenic phenotype observed in other *CDK12*-mutant cancers [58]. On the other hand, *CDK12* loss antagonizes *PTEN*-driven tumorigenesis, explaining their rare co-occurrence [10, 18]. In tumors chronically adapted to biallelic *CDK12* loss, most HR genes are transcriptionally restored, except for *ATM*, which remains truncated and downregulated. Consequently, such tumors retain functional HR and lack genomic scar signatures typical of HR-deficient cancers. This adaptation may explain the limited clinical benefit of PARP monotherapy in CDK12-mutant mCRPC. CDK13 appears to compensate for CDK12 loss, sustaining expression of DNA repair programs. Targeting CDK13 genetically or pharmacologically resensitizes *CDK12*-mutant models to transcriptional stress and DNA damage, both in vitro and in xenograft systems. These findings highlight CDK13 inhibition as a potential strategy to exploit vulnerabilities of *CDK12*-deficient prostate cancer [17].

Clinically, patients with *CDK12*-mutant mCRPC derive partial benefit from PARP + AR-pathway inhibitors, improving radiographic progression-free survival (HR 0.58, *p* = 0.029) but not overall survival [62]. New selective CDK12/13 inhibitors and degraders such as YJ9069 demonstrate efficacy in preclinical models of *CDK12*-deficient prostate cancer, suggesting opportunities for biomarker-guided therapeutic development [18].

### Breast cancer

The *CDK12* gene is located on chromosome 17q12, approximately 200 kb upstream of ERBB2 (HER2) oncogene, and is frequently co-amplified with HER2 in HER2^+^ breast cancers [11–13]. The TCGA project identified recurrent somatic alterations in *CDK12* (bi-allelic deletions, genomic amplifications and mutations) in 13% of breast cancers [52]. This co-amplification results in elevated *CDK12* mRNA, phosphorylation, and protein abundance, correlating with shorter progression-free survival [13, 63]. This amplification is also commonly found in breast cancer cell lines, suggesting a proliferative advantage of these cancer clones upon in vitro culture (Fig. 3). Functionally, CDK12 promotes tumor initiation, self-renewal, and trastuzumab resistance through activation of WNT/β-catenin and IRS1–ErbB–PI3K–AKT signaling [14]. *CDK12* overexpression enhances stem-like transcriptional programs and drives lapatinib resistance, whereas its depletion impairs tumorigenic capacity in vivo [13, 14]. Depletion of *CDK12* induces elongation defect in downregulated genes and increases the usage of intronic polyadenylation which give rise to truncated isoforms of long genes [63].

Co-amplified tumors exhibit strong dependency on CDK12 activity. CDK12 inhibition restores sensitivity to HER2-targeted therapy and suppresses downstream PI3K/AKT signaling [13]. Beyond HER2-positive disease, CDK12/CDK13 inhibition enhances apoptosis and reverses PARP-inhibitor resistance in triple-negative breast cancer (TNBC) by disrupting HR through RAD51 downregulation [40, 64]. In clinical settings, early-phase trials of broad CDK inhibitors such as dinaciclib demonstrated partial responses but dose-limiting toxicity [65]. Given the high frequency of *CDK12* amplifications in this population, clinical trials of new CCNK/CDK12/13 inhibitors should prioritize this patient group.

### Ovarian cancer

High-grade serous ovarian cancer (HGSOC) harbors *CDK12* mutations in approximately 9% and *CDK13* alterations in 2–3% of cases, defining a transcriptionally distinct subset characterized by defective expression of DNA damage response genes [21]. Loss or inhibition of CDK12 disrupts transcription of HR genes such as *BRCA1* and *ATR*, inducing a “BRCAness” phenotype characterized by replication stress and tandem duplications [15]. Pharmacologic inhibition with THZ-531 induces G₂/M arrest, apoptosis, and accumulation of unrepaired DNA lesions in HGSOC cell lines and patient-derived organoids [21]. Moreover, THZ-531 restores sensitivity to PARP inhibitors in previously resistant models, confirming a synthetic-lethal interaction between transcriptional elongation blockade and HR deficiency [21, 39]. Overall, CDK12/13 dysfunction defines a therapeutically exploitable transcriptional vulnerability in HGSOC that can be leveraged through combination strategies.

### Group 3 medulloblastoma

Group 3 Medulloblastoma (MB) is characterized by *MYC* amplification and poor prognosis [66]. Since MYC-driven tumors rely on transcriptional and splicing programs for survival, indirect targeting of MYC vulnerabilities through RNA processing inhibition has emerged as a promising strategy [67]. The covalent CDK12/13 inhibitor THZ-531 blocks Pol II CTD phosphorylation in MB, suppresses DDR, cell-cycle, and replication-related transcriptional programs, leading to extensive DNA damage and apoptosis in MYC-high MB cells [66].

Transcriptomic analyses show ~30% overlap between MYC- and THZ-531-regulated genes, confirming that CDK12/13 activity sustains MYC-dependent oncogenic transcription. Low-dose THZ-531 pretreatment sensitizes MYC-high MB cells to PARP inhibition, indicating synthetic lethality between transcriptional stress and DNA-repair blockade. These findings suggest that CDK12/13 maintain DDR gene expression essential for genome integrity in MYC-driven MB, and that their inhibition may therapeutically exploit MYC-induced vulnerabilities [66, 68, 69].

### Colorectal cancer

CDK12 expression is significantly upregulated in colorectal cancer (CRC), promoting malignancy through WNT/β-catenin and PI3K–AKT signaling pathways [30, 70]. Mutations and amplifications of CDK12 and CDK13 are prevalent in 5 to 10% of patients with CRC [71]. CDK12 knockdown or pharmacologic inhibition (THZ-531, SR-4835) reduces proliferation, clonogenicity, and invasiveness, while inducing apoptosis and G1 arrest [30]. In vivo, CDK12 inhibition decreases tumor burden and improves colitis-associated CRC histopathology [42]. Mechanistically, CDK12 inhibition suppresses β-catenin and its downstream targets and triggers autophagy via AKT/FOXO3, which enhances anti-PD-1 efficacy [70].

Functional overlap between CDK12 and CDK13 suggests a compensatory mechanism, in which CDK13 can partially sustain transcription of DNA-repair genes when *CDK12* is lost. Dual blockade of both kinases diminishes expression of long *BRCA1* transcript isoforms in CRC and markedly increases vulnerability to combined CDK12 and PARP inhibition [72].

Combining cyclin K/CDK12/CDK13 degrader, NCT02, with oxaliplatin and irinotecan exhibited synergistic anti-tumor effects in metastatic CRC cells [73]. Moreover, dinaciclib-mediated CDK12 inhibition increased radiosensitivity in colon cell lines [74].

In a retrospective analysis of metastatic CRC, patients harboring at least one pathogenic mutation in DDR genes (*n* = 11), including *CDK12*, demonstrated a significant improvement with FOLFOX/XELOX compared to FOLFIRI (3.4 vs. 1.8 y; *p* = 0.042). However, the number of patients with mutant *CDK12* was too small to draw statistically significant conclusions [75].

### Hepatocellular carcinoma

Transcriptomic analyses of TCGA datasets reveal significantly elevated *CDK12* expression in hepatocellular carcinoma (HCC) compared with non-tumor liver tissue, correlating with poorer overall survival [76]. Functional studies confirm that CDK12 is essential for HCC cell survival: knockdown or pharmacologic inhibition with THZ-531 reduces proliferation and induces apoptosis in Hep3B and Huh7 liver cancer cell lines, as well as in in vivo xenografts. Mechanistically, CDK12 inhibition downregulates DDR genes in HCC, leading to accumulation of unrepaired DNA lesions.

Combination treatment with sorafenib or ATM inhibitors markedly enhanced cytotoxicity, whereas minimal synergy was observed with inhibitors of PARP, ATR, or CHEK1. *CDK12* knockdown combined with sorafenib suppresses tumor growth by over 70% and reduces expression of EGFR, c-MET, and DDR1, key drivers of HCC progression [76]. These findings highlight CDK12 as a pro-survival transcriptional kinase that integrates oncogenic signaling and DNA-repair regulation in HCC, providing a rationale for dual targeting of CDK12 and receptor-tyrosine-kinase pathways.

### Ewing sarcoma

Ewing sarcoma is characterized by a fusion of the genes coding RNA-binding protein *EWS* and an *ETS* DNA-binding domain, most commonly *EWS/FLI1*. Genetic and pharmacologic suppression studies show that CDK12, not CDK13, is critical for the survival of Ewing sarcoma cells [20]. THZ-531 markedly downregulates HR and DDR genes, including *BRCA1, RAD51, FANCF, and XRCC2*, resulting in replication stress and apoptosis. Loss of *EWS/FLI1* confers resistance to THZ-531, confirming that the *CDK12* dependency is driven by the fusion-associated transcriptional program [20].

Combination therapy with THZ-531 and PARP inhibitor oolaparib produces strong synergy, impairing DNA-repair capacity and suppressing tumor growth in xenograft and patient-derived models without systemic toxicity. Supporting data from the Genomics of Drug Sensitivity in Cancer (GDSC) database identify *EWS/FLI* as a biomarker of sensitivity to DNA-damaging agents [77]. Collectively, these findings demonstrate that *EWS/FLI1*-driven tumors rely on CDK12-mediated DDR transcription, and that combined CDK12 and PARP inhibition represents a rational synthetic-lethal strategy in Ewing sarcoma [20].

### Osteosarcoma

CDK12 has been found as a metastatic vulnerability in osteosarcoma. Functional screening and pharmacologic inhibition studies show that THZ-531 suppresses colony formation, induces DNA damage, and promotes apoptosis in a dose-dependent manner across multiple human osteosarcoma cell lines [78]. In a murine osteosarcoma metastasis model, *CDK12* knockout markedly reduced lung metastases [78]. Broader inhibition with dinaciclib (SCH 727965), a pan-CDK inhibitor targeting CDK1/2/5/9/12, achieves disease control in osteosarcoma xenografts [79]. These findings suggest that CDK12 activity supports metastatic progression through maintenance of DNA-repair transcriptional programs and survival signaling, and that its inhibition may provide therapeutic benefit in advanced osteosarcoma.

### Gastric cancer

Immunohistochemical analysis by Ji et al. showed that approximately half of the examined gastric cancer cases (*n* = 43) exhibited high CDK12 expression, which correlated with shorter overall survival, diffuse-type histology, and increased lymph node metastasis [80]. Elevated CDK12 expression was independent of TNM stage but was enriched in aggressive subtypes, suggesting its potential role in tumor progression. Given the proximity of CDK12 to ERBB2 on chromosome 17q12, co-amplification events likely contribute to its overexpression in HER2-positive gastric cancers [14] (Fig. 3). Functionally, CDK12 is proposed to sustain oncogenic transcription via ErbB–PI3K–AKT and WNT pathways, mirroring its role in HER2^+^ breast tumors [14, 25]. While functional studies remain limited, *CDK12* knockdown decreases proliferation and migration in gastric cancer cell lines, supporting its classification as a potential prognostic marker and therapeutic target in molecularly defined gastric cancer.

### Non-small cell lung cancer

Larsen et al. demonstrated that CDK12 regulates the alternative processing of PD-L1 mRNA in non-small-cell lung cancer (NSCLC) cells. Although blockade of the PD-1/PD-L1 interaction has transformed NSCLC therapy, a substantial fraction of patients does not respond, partially due to heterogeneity in PD-L1 isoform expression. Membrane-bound PD-L1 (mPD-L1) promotes immune evasion by fostering an immunosuppressive tumor microenvironment, yet mPD-L1 positivity does not always predict therapeutic benefit. CDK12 controls this regulatory layer by governing alternative mRNA splicing and 3’ processing of PD-L1 transcripts.

Specifically, *CDK12* loss or inhibition with THZ-531 increases the ratio of the shorter PD-L1v4 isoform to the canonical PD-L1v1, thereby modulating PD-L1 responsiveness to interferon-γ stimulation [59]. These findings highlight CDK12 as a transcriptional regulator of immune checkpoint gene expression, although the broader tumor-intrinsic functions of CDK12/13 in NSCLC remain to be defined.

### Myeloid neoplasms

CDK13 plays a key role in regulating myeloid lineage differentiation, RNA processing, and megakaryocyte development in bone marrow cultures [81]. Analyses of DepMap identified cyclin K as essential across 62 hematological cancer cell lines [5]. Elevated CDK13 expression has been reported in myelodysplastic/myeloproliferative neoplasm with ring sideroblasts and thrombocytosis (MDS/MPN-RS-T), in which 80–90% of patients harbor SF3B1 mutations, a splicing factor regulated by CDK12/13 [82–84]. Through phosphorylation-dependent interactions with RNA polymerase II, CDK12/13 facilitate SF3B1-mediated co-transcriptional splicing, while pharmacologic inhibition with THZ-531 disrupts this complex [83]. SF3B1 mutations are also linked to increased thrombotic risk [82].

In acute myeloid leukemia (AML), aberrant transcriptional elongation is a hallmark feature [85]. Dual inhibition or genetic loss of CDK12/13 reduces RNA polymerase II elongation rate and processivity by ~50%, with the strongest effects observed in long multi-exonic genes. This treatment decreases Ser2 and Thr4 phosphorylation on the Pol II C-terminal domain, leading to premature transcription termination and altered alternative last-exon usage, generating shortened mRNA isoforms [5]. Consistently, *CDK12/13* knockout or knockdown impairs the growth of diverse myeloid leukemia cell lines. Most AML lines treated with THZ-531 exhibit IC₅₀ values between 34–116 nM, with FAB M4/M5 subtypes showing the greatest sensitivity. Moreover, venetoclax-resistant AML models, including primary patient samples, remain sensitive to CDK12/13 inhibitors (THZ-531, SR-4835), whereas normal hematopoietic stem and progenitor cells are largely spared at therapeutic doses [85]. Notably, acute myeloid and lymphoblastic leukemia cell lines show a specific dependency on *CDK13* (Fig. 3), warranting investigation into whether its biological function is more pronounced or distinct in acute leukemias.

### Melanoma

In BRAF-mutated melanoma, CDK12 is constitutively activated through phosphorylation by ERK1/2, a key effector of the RAS/MAPK signaling cascade [44]. This activation maintains transcription of long DDR genes while repressing stress-induced, short transcripts. Pharmacologic inhibition of CDK12 with THZ-531 reverses this transcriptional balance, downregulating DDR and cell-cycle genes while upregulating AP-1 and NF-κB targets, thereby promoting apoptosis and loss of tumor viability [44]. Combined inhibition of CDK12 and MAPK signaling produces synthetic lethality in melanoma cell lines and murine models, highlighting the potential of dual pathway targeting to overcome resistance to BRAF and MEK inhibitors. Similar to *CDK12* loss, *CDK13* mutations lead to the accumulation of short RNAs and prematurely terminated intronic RNAs, which can accelerate melanoma oncogenesis [86]. Given the high tumor mutational burden associated with skin cancer, further studies investigating if CDK12/13 mutations contribute to this genomic instability are warranted. Of note, both melanoma and non-melanoma skin cancers show exceptionally high frequencies of *CDK12* and *CDK13* mutations across all cancers (Fig. 3). Given the high tumor mutational burden characteristic of skin cancer, it is critical to determine whether these mutations directly contribute to this genomic instability. Furthermore, *CDK12* mutations in melanoma are already known to impact immunotherapy response [61] Analysis of Reduced CDK12/13 expression in metastatic melanoma patients significantly predicted improved survival and response to immune checkpoint blockade [61]. Treatment of melanoma B16–F10 cells with CDK12/13 PROTAC YJ1206 results in STING signaling activation. This effect was observed in prostate cancers and other solid tumor cell lines. Combined treatment with YJ1206 and anti-PD1 on subcutaneous melanoma tumor in FVB mouse models resulted in significant synergistic effect in comparison to therapy with a single compound or vehicle (*p* < 0.0001) [61].

## CDK12/13 inhibitors

The dysfunction of CDK12 and CDK13 has been linked to cancer progression and therapy resistance, making them promising targets for new therapeutic agents [20]. Drug development in this field has advanced rapidly, moving from broad-spectrum CDK inhibitors to highly specific molecules and targeted degraders with distinct mechanisms of action, creating a versatile toolkit for translational oncology.

### The rationale: inducing “BRCAness” and synthetic lethality

The therapeutic rationale for targeting CDK12 and CDK13 derives from their central role in maintaining genome integrity through transcriptional regulation of DNA-damage-response (DDR) genes. These kinases ensure expression of long genes critical for homologous recombination, including *BRCA1* and *ATR* [2]. Pharmacologic inhibition of CDK12/13 suppresses DDR gene transcription, producing a “BRCAness” phenotype in normally HR-proficient tumors [19]. This acquired HR deficiency creates a synthetic-lethal vulnerability, rendering cells dependent on compensatory repair pathways such as PARP-mediated repair. Consequently, CDK12/13 inhibition sensitizes tumor cells to PARP inhibitors and DNA-damaging agents, effectively expanding the utility of HR-directed therapies [15, 39].

### Evolving chemical modalities: from broad inhibition to targeted degradation

**Fig. 4:**
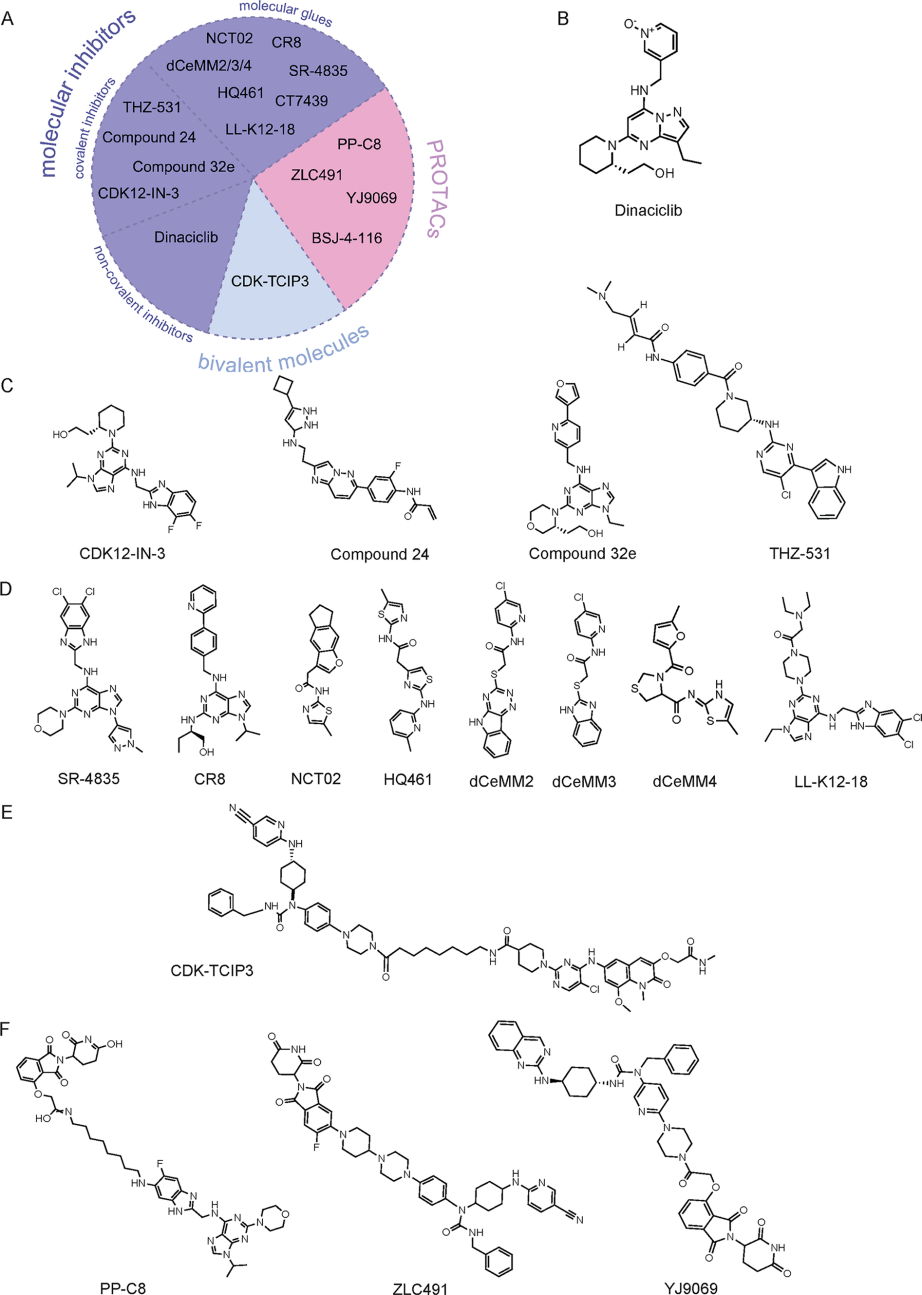
The evolving chemical landscape of CDK12/13-targeted therapies. (**A**) Overview of the major classes of CDK12/13-targeted agents. The field has progressed from (**B**) non-covalent and (**C**) covalent kinase inhibitors to newer chemical modalities, including (**D**) molecular glues and (**F**) PROTAC degraders that induce proteasomal degradation. (**E**) Bivalent molecules represent emerging chemical biology strategies linking CDK12/13 inhibition to transcriptional regulators

The development of CDK12/13-targeting agents has been a story of increasing precision, progressing from broad-spectrum ATP-competitive inhibitors to highly selective degraders and molecular glues that modulate protein stability rather than simply blocking catalytic activity (Fig. 4A). Early studies employed multi-CDK inhibitors such as flavopiridol and dinaciclib (Fig. 4B). While generally classified as pan-CDK inhibitor (targeting CDK1/2/5/9), dinaciclib has been shown to exhibit potent, direct inhibitory activity against CDK12 [40]. These compounds displayed promising antitumor effects but also dose-limiting toxicities and lack of specificity [87]. Subsequent research yielded more selective covalent inhibitors (Fig. 4C). A notable example is THZ-531, an ATP-site-directed covalent inhibitor that irreversibly binds CDK12/13 and synergizes with PARP inhibitors in models of hepatocellular carcinoma and Ewing sarcoma [20]. Other agents, such as CDK12-IN-3, have shown potent activity in combination with olaparib in ovarian cancer models [88].

Building on these advances, molecular glues and PROTAC degraders (Fig. 4D–F) achieve target selectivity by promoting ubiquitin-mediated degradation of the CDK12/13–cyclin K complex instead of conventional kinase inhibition. These approaches provide more durable suppression of CDK12/13 function and might prevent kinome rewiring or resistance-inducing mutations observed in conventional kinase inhibitors.

To contextualize this medicinal chemistry evolution, Table 1 summarizes the principal classes of CDK12/13-targeting compounds, outlining their pharmacologic mechanisms and preclinical or clinical performance. These diverse strategies allow fine-tuned dissection of CDK12/13 biology and offer multiple routes to pharmacologically induce “BRCAness,” enhance PARP-inhibitor sensitivity, and overcome transcriptional therapy resistance across cancer types (Table 1).

**Table 1:** Summary of CDK12/13 inhibitors and degraders

Compound class	Pharmacologic mechanism	Compound name	Preclinical/clinical highlights
*Non-covalent broad-spectrum CDK inhibitor*	Non-covalent ATP-competitive pan-CDK inhibitor (targets CDKs 1/2/5/9, also exhibits potent direct CDK12 activity)	Dinaciclib	Synergy with PARPi in breast cancer [44, 62], PFS of 481 days in CLL (independent of del17p13.1 status) [89], hematologic toxicity in TNBC trial (NCT01624441) [65]
*Covalent CDK12/13 inhibitor*	Covalent (irreversible), ATP-site–directed CDK12/13 kinase inhibitor (irreversible Cys-binding [90])	THZ-531	Synthetic lethality with PARPi in Ewing sarcoma [91]; Synergy with sorafenib in HCC [76] Synergy with oxaliplatin in gastric cancer xenograft model [92] Sensitization of prostate cancer cells to enzalutamide [93], increase anti-tumoral effect in androgen-sensitive prostate cancer cells [94] Inhibition of osteosarcoma spheroid formation and 2D cell colony, decreased osteosarcoma cell proliferation, diminished DNA repair [91] Induction of irreparable DNA damage in MYC-high Group 3 medulloblastoma cells, synergy with cisplatin and olaparib [66] Synergy with lapatinib, celarasertib, AZD7762 in high grade serous ovarian cancer [21] Impairment of SF3B1 (splicing factor) with chromatin [83] Diffuse large B-cell lymphoma and mantle cell lymphoma suppression in vitro [95]
		CDK12-IN-2 (Compound 24)	Potent against TNBC cell lines (EC50 ≈ 5–6 Nm) [96]
		CDK12-IN-3	Synergy with olaparib in ovarian cancer PDOs and PDXs; induces DNA double-strand breaks and NHEJ activity [88]
		CDK-IN-13 (Compound 32e)	Suppresses growth of HER2^+^ trastuzumab-sensitive and trastuzumab-resistant breast cancer cells [97]
*Molecular glue degraders*	Reversible (non-covalent) small molecules that act as molecular glue degrader and induce proximity between CDK12/13–Cyclin K complex and the DDB1–CUL4–RBX1 E3 ligase, promoting ubiquitin-mediated degradation [98]	SR-4835	Induces “BRCAness” phenotype, sensitizes to PARPi in TNBC cell lines and PDX models, orally bioavailable, tolerable in mice [30], impairs TNBC tumor growth as a single agent and in combination with cisplatin [19] Overcomes resistance to KRAS G12C inhibitor sotorasib in NSCLC preclinical models [99] Alleviates colitis-associated colorectal tumorigenesis, inhibits MC38 allograft outgrowth, and suppresses liver metastasis in C57BL/6 mice [30] Enhances the antitumor efficacy of *anti*-PD-1 Ab in CD8+ T cell-dependent manner in the 4T1 metastatic breast cancer model in BALB/c mice [100]
		NCT02	Synergy with oxaliplatin or irinotecan in metastatic CRC, esp. TP53-mutant [73]
		CR8	Downregulates MYC; inhibits neuroblastoma xenografts [101, 102]
		HQ461	Reduce Ser2 phosphorylation and DDR gene transcription; LL-K12-18 active in TNBC [103, 104]
		dCeMM2–4	
		LL-K12-18	Reduces mRNA levels of DDR genes & increases apoptosis in TNBC cell lines [103]
		CT7439	Active in OV-90 cells; synergy with PARPi (olaparib, niraparib) or carboplatin; entered Phase I clinical trials (NCT06600789) [105]
*PROTAC degrader*	Bifunctional chimeric molecules that simultaneously bind CDK12/13 and an E3 ligase (e.g., CRBN, VHL, or DDB1), forming a ternary complex that triggers selective ubiquitination and proteasomal degradation	ZLC491	Inhibits TNBC xenografts, suppresses expression of DDR genes; oral bioavailability of ~50% in rats [106]
		PP-C8	Downregulation of DDR gene expression; Synergistic with PARPi in TNBC cell line [107]
		YJ9069	Synergistic lethality with AKT inhibitors on PDX prostate cancer models [108]
		BSJ-4–116 (CDK12 specific degrader)	Causes premature cleavage and polyadenylation of DDR genes; synergistic lethality with olaparib on T-ALL cell lines [109]
*Dual target bivalent molecule*	Bifunctional molecule linking CDK12/13 to transcription factor BCL6 at chromatin	CDK-TCIP3	Induces cell death of DLBCL cells (EC_50_ ~ 609 nM after 72 h) with the 10 times greater effect than CDK12/13 inhibitor alone [110]

Summary of CCNK/CDK12/13-engaging compounds. The compounds are classified by pharmacologic mechanism, selectivity, and stage of preclinical or clinical development. Abbreviations: PARPi, poly(ADP-ribose) polymerase inhibitor; TNBC, triple-negative breast cancer; DLBCL, diffuse large B-cell lymphoma, NHEJ, non-homologous end joining DNA repair; NSCLC, non-small cell lung cancer; PDO, patient-derived organoid; PDX, patient-derived xenograft; PROTAC, proteolysis-targeting chimera; PD-1, programmed death 1

## Perspectives and conclusions

CDK12 and CDK13 have emerged as master regulators of the transcriptional landscape with profound implications for cancer biology and therapy. Their inhibition represents a paradigm shift in therapeutic strategy, moving beyond the blockade of single DNA-repair nodes to pharmacologically inducing a state of transcriptional “BRCAness”. Mechanistically, this phenotype arises through dual mechanisms. By crippling high-fidelity homologous recombination, CDK12/13 inhibition forces tumor cells to rely on error-prone repair pathways such as non-homologous end joining. In parallel, disruption of RNA polymerase II interactions with the core splicing factors, including SF3B1, promotes mis-splicing and premature termination of DDR transcripts. Together, these effects suppress genome-stability programs and create a potent synthetic-lethal vulnerability.

The biological and therapeutic complexity of CDK12/13 stems from their context-dependent functions. In some malignancies, notably HER2-positive breast and gastric cancers, *CDK12/13* amplification or overexpression acts as a transcriptional oncogenic driver that reinforces PI3K–AKT and WNT signaling. In such settings, CDK12/13 inhibitors directly suppress oncogenic transcriptional output, yielding clear therapeutic indication. In contrast, in tumors such as ovarian and metastatic prostate cancers, CDK12 loss-of-function mutations or deletions define a tumor-suppressor phenotype characterized by genomic instability and tandem duplications. These lesions are typically long-standing and subject to cellular adaptation: over time, cancer cells may partially restore HR competence, often through CDK13-mediated compensation or re-wiring of DNA-repair networks. This chronic adaptation explains why CDK12-mutant tumors frequently lack HR-deficiency genomic scars and show limited sensitivity to PARP monotherapy. Nevertheless, pharmacologic CDK12/13 inhibition in such chronically adapted cancers remains therapeutically relevant. Acute inhibition can re-create a transient transcriptional crisis, re-inducing DDR deficiency, replication stress, and apoptosis, thereby reinstating the BRCAness phenotype that had been functionally bypassed. This paradox highlights the importance of distinguishing oncogenic addiction from adaptive compensation when designing therapeutic strategies. Rather than being mutually exclusive, these dual roles of CDK12/13 illustrate how their inhibition can either directly suppress tumor-promoting transcription or exploit residual vulnerabilities in DNA-repair–adapted tumors.

Despite these complexities, the rapid development of next-generation inhibitors, degraders, and molecular glues has provided a powerful toolkit to dissect CDK12/13 biology with temporal precision. Future success will depend on resolving the functional divergence and overlap between these highly homologous kinases. A compelling example of this need is the recent work by *Frank et al.*, who found that while acute CDK12 loss induces the expected HR deficiency phenotype, metastatic prostate cancers adapted to chronic CDK12 biallelic loss largely regain HR competence [17]. Critically, these adapted tumors failed to respond to PARP monotherapy but demonstrated a novel vulnerability to targeting CDK13 by sgRNA or the dual CDK12/13 inhibitor SR-4835. This finding underscores that functional redundancy and adaptive compensation - specifically, the compensatory role of CDK13 following chronic CDK12 loss - are major determinants of therapeutic response. This critical role of CDK13 extends beyond compensation, as acute myeloid leukemia cell lines show a specific, non-redundant dependency on CDK13, suggesting it is a therapeutic target in certain hematologic malignancies, even conferring sensitivity to CDK12/13 inhibitors in venetoclax-resistant models. To resolve the functional divergence and overlap between these highly homologous kinases, a multi-omics approach can be considered. Quantitative phosphoproteomics such as whole proteomic and phospho-proteomic mass spectrometry could be used to map their distinct protein substrates, ChIP-sequencing may clarify their respective genomic binding sites and Pol II phosphorylation patterns, and long-read RNA sequencing could quantify their differential impacts on alternative splicing and premature polyadenylation.

The translational trajectory of CDK12/CDK13 inhibitors must be critically assessed against the repeated clinical setbacks encountered by previous CDK-targeting agents. Early, broad-spectrum CDK inhibitors, such as flavopiridol and dinaciclib, were largely derailed by severe, dose-limiting toxicities (e.g., myelosuppression) due to their indiscriminate inhibition of cell cycle (CDK1/2) and global transcriptional (CDK9) programs. Subsequent success with highly selective CDK4/6 inhibitors in HR+ breast cancer demonstrated that specificity and biomarker-driven patient selection are paramount. CDK12/CDK13 inhibitors are strategically positioned to overcome these historical limitations through two key design advantages: first, their high selectivity minimizes the off-target transcriptional shut-down that caused toxicity in older pan-CDK agents; and second, the clinical strategy is anchored in exploiting a synthetic lethal vulnerability. By targeting tumors defined by CDK12 loss of function mutations, these agents bypass the challenge of unselected populations and leverage a specific DDR deficiency. This shift from aiming for broad tumor suppression to exploiting known molecular frailties represents the most realistic pathway for CDK12/CDK13 selective agents to achieve enduring clinical relevance. Ultimately, selectively dismantling the transcriptional programs governed by CDK12 and CDK13 offers a powerful means to undermine oncogenic resilience and expand the therapeutic reach of precision oncology.

## Electronic supplementary material

Below is the link to the electronic supplementary material.

Supplementary Material 1

## Data Availability

No datasets were generated or analysed during the current study.

## Abbreviations

AKT: Protein kinase B
AML: Acute myeloid leukemia
AP-1: Activator protein-1
AR: Androgen receptor
ARPi: Androgen-receptor pathway inhibitor
ATM: Ataxia telangiectasia mutated
ATR: ATM- and Rad3-related protein
BCL6: B-cell lymphoma 6
BRCA: Breast cancer gene (BRCA1/BRCA2)
CDK: Cyclin-dependent kinase
CHK1: Checkpoint kinase 1
CRBN: Cereblon (E3-ligase substrate receptor)
CRISPR: Clustered regularly interspaced short palindromic repeats
CRC: Colorectal cancer
CTD: C-terminal domain
CUL4: Cullin-4 (E3-ligase scaffold)
DDB1: DNA-damage binding protein 1 (E3-ligase adaptor)
DDR: DNA damage response
DLBCL: Diffuse large B-cell lymphoma
EGFR: Epidermal growth factor receptor
ERBB2 (HER2): Receptor tyrosine kinase also known as HER2
ERK: Extracellular signal-regulated kinase
EWS/FLI1: Ewing sarcoma breakpoint region 1/Friend leukemia integration 1 fusion
FAB: French-American-British classification
FANCD2/FANCI: Fanconi anemia complementation group D2/I
GDSC: Genomics of Drug Sensitivity in Cancer
HCC: Hepatocellular carcinoma
HGSOC: High-grade serous ovarian cancer
HR: Homologous recombination
IC50: Half-maximal inhibitory concentration
IPA: Intronic polyadenylation
MAPK: Mitogen-activated protein kinase
mCRPC: Metastatic castration-resistant prostate cancer
NF-κB: Nuclear factor kappa-light-chain-enhancer of activated B cells
NHEJ: Non-homologous end joining
NSCLC: Non-small-cell lung cancer
OS: Overall survival
PARP: Poly(ADP-ribose) polymerase
PD-1 / PD-L1: Programmed cell death-1/programmed death-ligand 1
PDO: Patient-derived organoid
PDX: Patient-derived xenograft
PFS: Progression-free survival
PIC: Pre-initiation complex
PI3K: Phosphoinositide 3-kinase
Pol II: RNA polymerase II
PROTAC: Proteolysis-targeting chimera
PSMA: Prostate-specific membrane antigen
p-TEFb: Positive transcription elongation factor b
RBX1: RING-box protein 1 (E3-ligase component)
SF3B1: Splicing factor 3B subunit 1
snRNP: Small nuclear ribonucleoprotein
TNBC: Triple-negative breast cancer
VHL: Von Hippel–Lindau (E3-ligase substrate receptor)
WNT: Wingless/Integrated pathway
